# Supplementary material for: Venues and segregation: A revised Schelling model
Source: PLoS One. 2021 Jan 22;16(1):e0242611. doi: 10.1371/journal.pone.0242611 (PMC7822376; doi:10.1371/journal.pone.0242611)
Supplement: S1 Appendix — (DOCX) [file pone.0242611.s009.docx]

# S1 Appendix: Complete settings for each study

| 1 |  |  |  |
| --- | --- | --- | --- |
| Group 1 |  | Group 2 |  |
| Tolerance | 0 to 1 | Tolerance | 0 to 1 |
| Density | 85 | Density | 85 |
| Exclusivity | 1 | Exclusivity | 1 |
| Obligatoriness | 1 | Obligatoriness | 1 |
| Number Venues | 1 | Number Venues | 1 |
| Neighborhood Dist. | 2 | Neighborhood Dist. | 2 |
| Venue Travel Dist. | 0 to 50 | Venue Travel Dist. | 0 to 50 |
| 2.1 |  |  |  |
| Group 1 |  | Group 2 |  |
| Tolerance | 0 to 1 | Tolerance | 0 to 1 |
| Density | 85 | Density | 85 |
| Exclusivity | 1 | Exclusivity | 1 |
| Obligatoriness | 1 | Obligatoriness | 1 |
| Number Venues | 5 | Number Venues | 5 |
| Neighborhood Dist. | 2 | Neighborhood Dist. | 2 |
| Venue Travel Dist. | 0 to 50 | Venue Travel Dist. | 0 to 50 |
| 2.2 |  |  |  |
| Group 1 |  | Group 2 |  |
| Tolerance | 0 to 1 | Tolerance | 0 to 1 |
| Density | 85 | Density | 85 |
| Exclusivity | 1 | Exclusivity | 1 |
| Obligatoriness | 1 | Obligatoriness | 1 |
| Number Venues | 8 | Number Venues | 8 |
| Neighborhood Dist. | 2 | Neighborhood Dist. | 2 |
| Venue Travel Dist. | 0 to 50 | Venue Travel Dist. | 0 to 50 |
| 3 |  |  |  |
| Group 1 |  | Group 2 |  |
| Tolerance | 0 to 1 | Tolerance | 0 to 1 |
| Density | 85 | Density | 85 |
| Exclusivity | 0 to 1 | Exclusivity | 1 |
| Obligatoriness | 1 | Obligatoriness | 1 |
| Number Venues | 3 | Number Venues | 3 |
| Neighborhood Dist. | 2 | Neighborhood Dist. | 2 |
| Venue Travel Dist. | 10 | Venue Travel Dist. | 10 |
